# Assessment of humoral immune response to different COVID-19 vaccines in patients undergoing maintenance hemodialysis

**DOI:** 10.1590/2175-8239-JBN-2022-0184en

**Published:** 2023-08-11

**Authors:** Ayman Abd El-Hameed, Mohammed Fouad Ahmed, Ali Omar Ahmed Ehmemeed, Ahmad Mokhtar, Walid Ahmed Ragab Abdelhamid

**Affiliations:** 1 Zagazig University, Faculty of Medicine, Internal Medicine Department, Zagazig, Egypt.; 2 Zagazig University, Faculty of Medicine, Clinical Pathology Department, Zagazig, Egypt.

**Keywords:** SARS-CoV-2, Immunoglobulin G, Renal Dialysis, BNT162 Vaccine, ChAdOx1 nCoV-19, SARS-CoV-2, Imunoglobulina G, Diálise Renal, Vacina BNT162, ChAdOx1 nCoV-19

## Abstract

**Introduction:**

The immune response to different Coronavirus Disease 2019 (COVID-19) vaccines is under-investigated in end-stage kidney disease (ESKD) patients, especially in the Middle East and North Africa. We carried out this research to estimate the effectiveness of COVID-19 immunization in ESKD patients on regular hemodialysis (HD).

**Methods:**

In this prospective observational study, we enrolled 60 ESKD patients on regular HD who had completed COVID-19 vaccination and 30 vaccinated healthy participants. Serum levels of severe acute respiratory syndrome coronavirus 2 immunoglobulin G (SARS-COV2 IgG) were quantified 1 month after completing the vaccination schedule, and all participants were followed up from October 2021 to March 2022. The vaccines used in the study were from Pfizer-BioNTech, AstraZeneca, and Sinopharm.

**Results:**

The median level of SARS-COV2 IgG was lower in HD patients than in healthy participants (p < 0.001). Regarding the type of COVID-19 vaccination, there was no statistical difference in SARS-COV2 IgG levels among HD patients. During the observation period, none of the HD patients had COVID-19.

**Conclusion:**

COVID-19 vaccination appeared to be protective in HD patients for 6 months and the side effects of vaccines were tolerable.

## RESUMO

**Introdução:**

A resposta imune a diferentes vacinas contra a doença do coronavírus 2019 (COVID-19) é pouco investigada em pacientes com doença renal em estágio terminal (DRET), especialmente no Oriente Médio e norte da África. Realizamos esta pesquisa para estimar a eficácia da imunização contra a COVID-19 em pacientes com DRET em hemodiálise regular (HD).

**Métodos:**

Nesse estudo observacional prospectivo, inscrevemos 60 pacientes com DRET em HD regular que haviam concluído o esquema de vacinação contra a COVID-19 e 30 participantes saudáveis vacinados. Os níveis séricos de imunoglobulina G da síndrome respiratória aguda grave do coronavírus 2 (SARS-COV2 IgG) foram quantificados um mês após a conclusão do esquema vacinal, e todos os participantes foram acompanhados de outubro de 2021 a março de 2022. As vacinas utilizadas no estudo eram da Pfizer-BioNTech, AstraZeneca e Sinopharm.

**Resultados:**

O nível mediano de SARS-COV2 IgG foi menor em pacientes em HD do que em participantes saudáveis (p < 0,001). Com relação ao tipo de vacinação contra a COVID-19, não houve diferença estatística nos níveis de SARS-COV2 IgG entre pacientes em HD. Durante o período de observação, nenhum dos pacientes em HD teve COVID-19.

**Conclusão:**

A vacinação contra a COVID-19 pareceu ser eficaz na proteção de pacientes em HD por 6 meses e os efeitos colaterais das vacinas foram toleráveis.

## Introduction

The outbreak of coronavirus disease-2019 (COVID-19) has been a global health catastrophe with a high incidence of complications, particularly in end-stage kidney disease (ESKD)^1,2^. Thus, vaccination against COVID-19 is important in patients with ESKD. Nevertheless, patients with ESKD are more susceptible to weakened immunological responses to pathogens and active immunization^3^. Various vaccines have been developed to combat COVID-19. Patients with ESKD cannot receive vaccines with live attenuated viruses as they are generally immunocompromised. Both mRNA vaccines and replication-defective viral-vectored vaccines are believed to be acceptable for administration to these patients^4^. The immune response to different COVID-19 vaccines is under-investigated in patients with ESKD, especially those on regular hemodialysis (HD), especially in the Middle East and North Africa. We carried out this study to evaluate the effectiveness of the COVID-19 vaccine in patients undergoing regular hemodialysis.

## Methods

This research was conducted at Zagazig university hospital. The study protocol followed Helsinki regulations and was approved by the Institutional Review Board of the Ethical Committee of Zagazig University (ZU-IRB #8038). A written informed consent was obtained from all participants. This prospective observational study included the following groups:

Group 1: Sixty ESKD patients on maintenance hemodialysis who received two doses of COVID-19 vaccine.

Group 2: Thirty age/sex-matched vaccinated healthy participants.

We excluded patients with acute kidney injury, history of COVID-19 infection, or active rheumatologic disease who were on immunosuppression, and kidney transplant recipients with failing allograft who still received immunosuppressive medications. Each participant underwent a medical history review and a comprehensive clinical assessment including age, sex, smoking, obesity, history of comorbid diseases, underlying cause of ESKD, hemodialysis duration, history of COVID-19 infection before vaccination, and side effects of vaccination. Investigations consisted of serum albumin, complete blood count (CBC), blood urea, total protein, serum creatinine, C-reactive protein, serum uric acid, serum electrolytes, serum ferritin, serum parathyroid hormone, and Kt/V, which was calculated using the Daugirdas^5^ formula. For all HD patients in this study, Kt/V was >1.2%. We measured antibodies against epitopes in the nucleocapsid (N) and spike (S) regions of COVID-19 virus (DRG Instruments, Germany). Serological testing was performed one month after completion of the vaccination schedule. The cut-off value was 50 AU/mL. The COVID-19 vaccines used for both patients and healthy participants in this study were from Pfizer-BioNTech, AstraZeneca, and Sinopharm. Pfizer-BioNTech is administered in the upper arm as an intramuscular injection in two doses, with the second dose 21 days after the first. The AstraZeneca vaccine uses a harmless, weakened animal virus (called a viral vector) that contains the genetic code for the coronavirus spike protein. Two doses of the AstraZeneca vaccine were given in the upper arm as intramuscular injection 21 days after the first. The AstraZeneca vaccine uses a harmless, weakened animal virus (called a viral vector) that contains the genetic code for the coronavirus spike protein. Two doses of the AstraZeneca vaccine were given in the upper arm as intramuscular injection 12 weeks apart. Sinopharm is an inactivated vaccine that is administered as an intramuscular injection in the upper 12 weeks apart. Sinopharm is an inactivated vaccine that is administered as an intramuscular injection in the upper arm. Two doses were administered 3 weeks apart.

All subjects were followed up for new COVID-19 infections from October 2021 to March 2022.

### Statistical Analysis

SPSS Version 23 was used to analyze the research data. Numerical values are presented as mean ± standard deviation (SD) and were examined using the T-test if normally dispersed or as median (range) and assessed using the Mann-Whitney U test if non-normally dispersed. Categorical variables are reported as number (percentages) and the chi-square test or Fisher’s exact test were used to compare the percentages between groups. The Kruskal-Wallis test was used to compare more than two groups of non-normally distributed data. Correlations between various study variables were assessed using Spearman’s correlation coefficient. Statistical significance was defined as a p-value of 0.05 or below.

## Results

Sixty-one percent of HD patients were males, and the mean age was 55.2 ± 12.7 years. In terms of age and sex, there were no statistically significant differences between HD patients and healthy participants (Table 1).

**Table 1:** Comparison between study groups regarding demographic data

	HD group (n = 60)	Control group (n = 30)	χ^2^	p-value
Age in years			T	
• Mean ± SD	55.2 ± 12.7	51.1 ± 8.5	1.81	0.074
Sex				
• Males	37 (61.7%)	14 (46.7%)	1.83	0.178
• Females	23 (38.3%)	16 (53.3%)		
Smoking	18 (30.0%)	9 (30.0%)	0	1
Obesity	12 (20.0%)	11(36.7%)	2.9	0.087
Duration of HD - median (range) in years	6.5 (0.5-30)	–	–	–
Underlying cause of ESKD		–	–	–
Interstitial nephritis	8 (13.3%)			
Polycystic kidney disease	4 (6.7%)			
Diabetes Mellitus	17 (28.3%)			
Hypertension	22 (36.6%)			
Preeclampsia	3 (5.0%)			
FSGS	3 (5.0%)			
Obstructive uropathy	3 (5.0%)			

SD = standard deviation, T = test of significance, χ^2^ = chi square test, FSGS = Focal segmental glomerulosclerosis.

HD duration was 0.5 to 30 years, with a median of 6.5 years. The main causes of ESKD were hypertension (36.6%) and diabetes mellitus (28.3%) (Table 1). Table 2 shows the laboratory data of the studied HD patients.

**Figure 1.**
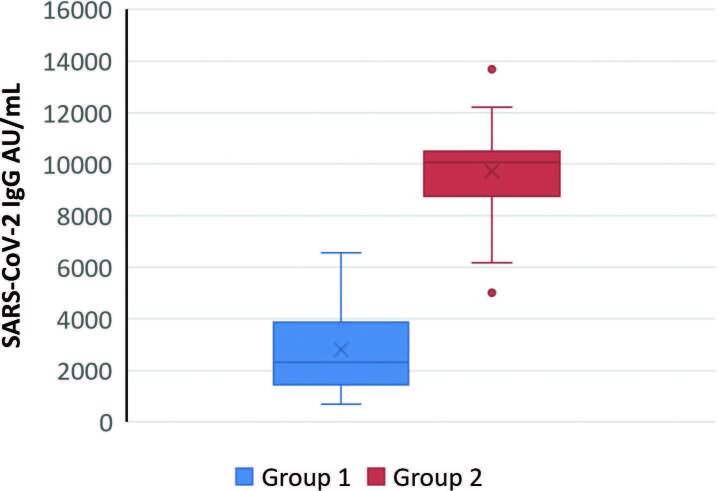
Medians and ranges of serum SARS COVID-19 IgG levels in HD patients (Group 1) and control group (Group 2) (p < 0.001).

**Figure 2.**
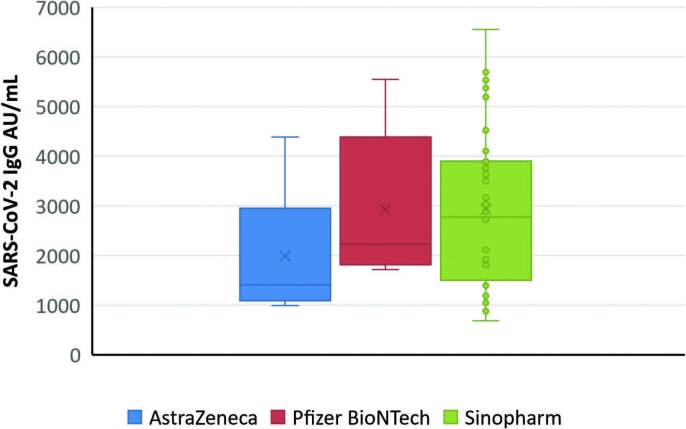
Medians and ranges of serum SARS COVID-19 IgG levels in HD patients according to the type of COVID-19 vaccine (p > 0.05).

All patients and healthy participants developed positive immune response. The median serum level of severe acute respiratory syndrome coronavirus 2 immunoglobulin G (SARS-COV2 IgG) in HD patients was considerably lower than in the healthy participants (p < 0.001) (Figure 1). No significant difference was observed in the levels of serum SARS-COV2 IgG according to the type of COVID-19 vaccine among HD patients (p > 0.05) (Figure 2).

**Table 2:** Laboratory data of HD patients

Variables	HD patients (n = 60) Mean ± SD
kt/v (%)	1.3 ± 0.11
Hemoglobin (gm/dL)	10.3 ± 1.7
WBC (mm^3)^	6.4 ± 2.2
Lymphocytes (mm^3)^	1.7 ± 0.63
Platelets (mm^3)^	242 ± 76.7
Blood Urea Nitrogen (mg/dL)	67.5 ± 14.8
Serum creatinine (mg/dL)	11.1 ± 2.9
Serum sodium (mmol/L)	131.2 ± 2.9
Serum potassium (mmol/L)	4.45 ± 0.61
Serum calcium (mg/dL)	8.7 ± 0.88
Serum phosphorus (mg/dL)	5.02 ± 1.3
Serum uric acid (mg/dL)	6.9 ± 1.3
Serum total proteins (gm/dL)	6.9 ± 0.72
Serum albumin (gm/dL)	3.9 ± 0.55
Serum ferritin (ng/mL)	343.1 ± 366.5
Parathyroid hormone (pg/mL)	454.1 ± 499.9
CRP (mg/L)	8.8 ± 10.9

SD = standard deviation, CRP = C-Reactive Protein.

We found no statistical difference between HD patients and healthy participants regarding the incidence of adverse effects of COVID-19 vaccines, and we didn’t detect any serious adverse effects such as anaphylaxis, thrombosis with thrombocytopenia syndrome, and Guillain-Barré syndrome (Table 3).

**Table 3:** Comparison between HD patients and control group regarding COVID-19 vaccine side effects

Variables	HD patients (n = 60)		Control group (n = 30)		χ^2^	P
	n.	%	n.	%		
Fatigue	16	26.7	11	36.7	0.95	0.33
Fever	18	30	12	40	0.9	0.34
Pain	3	5	–	–	f	0.55
Arthralgia	2	3.3	–	–	f	0.99
Headache	1	1.7	1	3.3	f	0.99

χ^2^ = chi square test, f = Fisher’s exact test.

There was no significant difference in serum SARS-COV2 IgG level among HD patients in relation to demographic parameters (p > 0.05), except for age. Mean serum IgG level was significantly higher in HD patients younger than 60 years than in HD patients >60 years (p = 0.001) (Table 4 ).

**Table 4:** Comparison of serum SARS-COV2 IgG level between HD patient and control group regarding demographic characteristics

Variables	HD patients (n = 60)	Control group (n = 30)	U	P
Age per years				
<60	3663 (1035–6558)	10042 (5018–13680)	6.2	0.0001
≥60	1936 (682–4522)	10065 (9978–12216)	2.8	0.005
U	3.4	0.36		
P1	0.001	0.89		
Sex				
Males	2176 (682-6558)	10292.5 (7384–13680)	5.5	0.0001
Females	2734 (987-5697)	9821 (5018–13680)	5.2	0.0001
U	0.11	2		
P1	0.91	0.04		
Obesity				
Obese	2080 (987-4385)	10060 (6178–13680)	4.1	0.0001
Non obese	2728 (682-6558)	10042 (5018–12216)	6.2	0.0001
U	0.81	0.32		
P1	0.42	0.75		

U = Mann-Whitney U, p > 0.05 insignificant, p < 0.05 significant, p < 0.001 highly significant, (p: comparison between HD patients and control group), (p1: comparison within group).

There was a significant negative correlation between serum SARS-COV2 IgG level and age and duration of HD. However, there was a positive correlation between serum SARS-COV2 IgG level and kt/v (%) in HD patients (Table 5).

**Table 5:** Correlations between serum SARS-COV2 IgG level and demographic and laboratory data in HD patients

Variables	Serum SARS-COV2 IgG (AU/mL) among HD patients (n = 60)	
	R	P
Age (per year)	–0.510	0.0001**
Duration of HD (in years)	–0.291	0.024*
kt/v (%)	+0.726	0.0001**
Hemoglobin (gm/dL)	+0.14	0.314
WBC (mm^3^)	+0.073	0.58
Lymphocytes (mm^3^)	+0.124	0.345
Platelets (mm^3^)	+0.033	0.799
Blood urea Nitrogen (mg/dL)	+0.044	0.736
Serum creatinine (mg/dL)	+0.077	0.559
Serum sodium (mmol/L)	–0.215	0.098
Serum potassium (mmol/L)	+0.012	0.925
Serum calcium (mg/dL)	–0.034	0.798
Serum phosphorus (mg/dL)	–0.039	0.766
Serum uric Acid (mg/dL)	–0.045	0.734
Serum total Protein (gm/dL)	+0.123	0.349
Serum albumin (gm/dL)	–0.159	0.224
Serum ferritin (ng/mL)	+0.105	0.425
Parathyroid hormone (pg/mL)	–0.148	0.26
C Reactive Protein (mg/L)	+0.206	0.115

(r) correlation coefficient,

^**^Correlation is significant at the 0.01 level (2-tailed),

^*^Correlation is significant at the 0.05 level (2-tailed).

## Discussion

COVID-19 has led to higher death and morbidity rates among patients on maintenance HD^3^. Furthermore, HD patients have an increased risk of developing serious COVID-19 infection-related consequences and have poor outcomes including higher risk of hospitalization, ICU stay, and mechanical ventilation^6^.

Internationally, SARS-CoV-2 immunization programs prioritize patients undergoing dialysis for immunization. CKD reduces the immune response to active infection and various vaccines, as reflected in the immune response to hepatitis B vaccine. Therefore, higher vaccine doses or timing changes are often required for these patients^7^. Several SARS-CoV-2 vaccines have been approved for the general population. HD patients should not receive live attenuated vaccines due to their immunocompromised status. The replication-defective viral-vectored vaccines and mRNA vaccines are thought to be suitable for administration to patients receiving maintenance HD treatment^4^. Few studies have examined the immune reaction to the COVID-19 vaccine in patients receiving maintenance HD. Therefore, the purpose of this study was to estimate the acquired immunity that develops in HD patients in response to different types of COVID-19 vaccines.

Our major observation was that patients undergoing maintenance HD had a favorable immune response post-vaccination, but was considerably lower than in healthy participants. Additionally, during the follow-up period, none of the vaccinated HD patients developed infection by COVID-19 (by clinical presentation or COVID-19 PCR). Our findings are consistent with those of Grupper et al.^8^, Attias et al.^9^, and Fucci et al.^10^ who reported that 96%, 86%, and 76%, respectively, of dialysis patients had positive immune response after vaccination with 2 doses of COVID-19 vaccines. Additionally, Fucci et al.^10^ found that the acquired immunity improved significantly following the third dose of the vaccine (97%). Earlier research on the immunological response in HD patients discovered an encouraging SARSCoV-2 spike protein immune reaction, but lower than in the non-dialysis cohort^11,12^. In addition, Simon et al.^13^ indicated that HD patients developed a weak humoral immune response three weeks after vaccination. Some studies, on the other hand, have demonstrated superior production of antibodies in response to COVID-19 vaccination in patients undergoing long-term dialysis, with 95% seroconversion rate^14,15,16^. The probable reason for the disparities in the aforementioned conclusions is the limited number of participants in some of the clinical trials.

Our HD patients were given different vaccines (AstraZeneca, Pfizer-BioNTech, and Sinopharm), but the median IgG titers did not vary significantly by vaccine type. This is in accordance with the Anand et al.^12^ study, which reported that the type of vaccine did not significantly affect median IgG titers.

HD patients over the age of 60 had a substantially decreased immune response than patients under the age of 60. Additionally, a longer dialysis duration was linked to a weakened response to COVID-19 vaccination in our study. On the other hand, effective dialysis dose was associated with a good immune response. In line with our results, Frantzen et al.^17^ reported that the elderly showed a poor antibody response and Anand et al.^12^ found that longer duration of dialysis and hypoalbuminemia were linked to a weak immune response to COVID-19 vaccination. Because aged T cells create short-lived inflammatory effector T cells rather than memory or follicular helper T cells, the effects of age on immune response can be associated with a reduction in immunologic memory with age^18^. Additionally, longer dialysis duration had a negative effect on adaptive immune response due to its cumulative impact on the health status of patients with ESKD (i.e., chronic inflammation, malnutrition, sarcopenia, and/or frailty)^19^. However, some studies found no associations between immune response and demographic variables such as age, sex, and body mass index^20,21,22^. This difference can be attributed to limited sample sizes, selection biases, and different population ethnicities.

In this study, we assessed the immune response to different vaccines including Sinopharm, Pfizer-BioNTech, and AstraZeneca. Of previous clinical trials, only Husain et al.^23^ studied the effects of Pfizer-BioNTech and Moderna mRNA-1273 vaccines in kidney transplant patients and Anand et al.^12^ studied the effects of Moderna, Johnson & Johnson, and Pfizer-BioNTech vaccines. All other studies only assessed the Pfizer-BioNTech vaccine.

Limitations of the study include that baseline antibody titers were not measured before vaccination. Thus, the serological response may indicate a previous asymptomatic infection. Additionally, the effect of vaccination on cellular immunity was not studied.

## Conclusion

After receiving two COVID-19 vaccine doses, patients on maintenance HD had a positive immune response for 6 months. The protective effect of the immune response was tolerated without significant side effects of vaccination. Booster doses of the COVID-19 vaccine may enhance the immune response in HD patients and are therefore recommended.
